# Close of the Third Volume of the Review

**Published:** 1859-11

**Authors:** 


CLOSE OF THE THIRD VOLUME OF THE REVIEW.—The present number terminates the third year of our editorial labors. In reviewing those labors we have every reason to be satisfied with their results. The Journal has gradually and steadily increased in usefulness and reputation, and has become one of the authoritative periodicals of the day, its pages being constantly referred to by all the leading journals in the United States and Europe. It has been the ardent desire of the editors to conduct the Review in such a manner as to render it worthy of the present enlightened state of the medical profession, and of the rising condition of our national medical literature, of which they have always been warm advocates. If, in their notices of native works, they have sometimes been obliged to assume the character of severe critics, they have the consolation to believe that their judgment has been generally approved by their professional brethren. They have never allowed themselves to be biased, in anything that has been thus done, by sectional feeling or jealousy. Their great endeavor has been to do justice to all; neither, on the one hand, to indulge in blind praise, nor, on the other, in unmerited condemnation. While their course has been one of great independence, leading sometimes to a bold expression of opinion, they have the gratification to know that the pages of the Review have never been sullied by one solitary personality. To their collaborators, who have so nobly sustained them in their efforts to build up an able and useful periodical, the editors tender their warmest acknowledgments, and confidently appeal to them for future support.

